# Physical restraint use and older patients’ length of hospital stay

**DOI:** 10.1080/21642850.2014.881258

**Published:** 2014-02-05

**Authors:** Xue Bai, Timothy C.Y. Kwok, Isaac N. Ip, Jean Woo, Maria Y.P. Chui, Florence K.Y. Ho

**Affiliations:** ^a^ Department of Applied Social Sciences, The Hong Kong Polytechnic University, Hung Hom, Kowloon, Hong Kong, People's Republic of China; ^b^ Jockey Club Centre for Positive Ageing, Shatin, New Territories, Hong Kong SAR, People's Republic of China; ^c^ Department of Medicine and Therapeutics, The Chinese University of Hong Kong, Shatin, New Territories, Hong Kong SAR, People's Republic of China; ^d^ Shatin Hospital & Bradbury Hospice, Shatin, New Territories, Hong Kong SAR, People's Republic of China

**Keywords:** physical restraint, older patients, length of hospital stay, nursing practice

## Abstract

In both acute care and residential care settings, physical restraints are frequently used in the management of patients, older people in particular. Recently, the negative outcomes of physical restraint use have often been reported, but very limited research effort has been made to examine whether such nursing practice have any adverse effects on patients' length of stay (LOS) in hospitals. The aim of this study was to examine the association between physical restraint use on older patients during hospitalization and their LOS. Medical records of 910 older patients aged 60 years and above admitted to one of the Hong Kong public hospitals in 2007 were randomly selected and recorded during July to September 2011. The recorded items included patients' general health status, physical and cognitive function, the use of physical restraints, and patients' LOS. Hierarchical regression analysis was conducted to analyze the data. The results indicated that older patients' general health status, physical, and cognitive function were important factors affecting their LOS. Independent of these factors, the physical restraint use was still significantly predictive of longer LOS, and these two blocks of variables together served as an effective model in predicting older patients' LOS in the hospital. Since physical restraint use has been found to be predictive of longer hospital stay, physical restraints should be used with more caution and the use of it should be reduced on older patients in the hospital caring setting. All relevant health care staff should be aware of the negative effects of physical restraint use and should reduce the use of it in hospital caring and nursing home settings.

Older patients are often put on physical restraints in nursing homes and during hospital stay (Burton, German, Rovner, Brant, & Clark, 1992; Huizing, Hamers, de Jonge, Candel, & Berger, 2007; Kwok, Mok, Chien, & Tam, 2006; Pellfolk, Gustafson, Bucht, & Karlsson, 2010; Wang & Moyle, 2005). Physical restraint refers to any equipment attached to or adjacent to a person's body that restricts the person's freedom of movement deliberately, and usually cannot be easily controlled or removed by the person (Retsas, 1998). Different types of restraints are used in clinical practice as a measure of patient management to ensure the safety of patients and staff (Hamers & Huizing, 2005), facilitate treatment (Lee, Chan, Tam, & Yeung, 1999), and compensate for understaffing (Evans & FitzGerald, 2002; Minnick, Mion, Leipzig, Lamb, & Palmer, 1998).

The use of physical restraints on older patients gives rise to a number of ethical issues. It has been suggested that physical restraints hinder the promotion of self-reliance in older people and disrespect older people's autonomy and dignity (Wang & Moyle, 2005). Hospital patients have expressed their feelings of lack of empathy following the use of physical restraints, and complained that they felt powerless and uncertain when being physically restrained (Chien, Chan, Lam, & Kam, 2005). Furthermore, it was found that patients who had been physically restrained during hospitalization were more likely to report nightmares and avoidance responses (Mohr, Mahon, & Noone, 1998), and they seemed to continue to experience these negative effects of physical restraints five years later (Mohr & Pumariega, 2001). Although physical restraints have always been thought as a preventive intervention to wandering and falls, studies have found that the use of bedrails did not reduce the likelihood of falls and injuries (Capezuti, Maislin, Strumpf, & Evans, 2002), while the removal of restraints made no change in the fall rate, injury rate as well as therapy disruptions (Hanger, Ball, & Wood, 1999; Kramer, 1994; Kwok et al., 2006; Lever, Molloy, Bedard, & Eagle, 1995; Levine, Marchello, & Totolos, 1995; Mion et al., 2001; Tilly & Reed, 2008).

In spite of the above issues, the use of physical restraints is still common in caring for older people in the hospital setting in Hong Kong (Yan, Kwok, Lee, & Tang, 2009). Previous research has chiefly focused on the effect of physical restraint use in reducing fall rate. While it is reasonable to investigate the effect of an intervention with respect to its aims and purposes, it is argued that other possible meaningful relationships such as its effect on length of stay (LOS) might have been overlooked.

LOS in hospital can be considered as a reasonable estimate of resource use in inpatient care as well as treatment outcomes (Brownell & Roos, 1995; Jiménez, Lam, Marot, & Delgado, 2004). Not only is excess LOS cost-ineffective in terms of the resource utilization (Wright et al., 2003), but also attributable to medical injuries during hospitalization and isolation of patients from their social network (Zhan & Miller, 2003). A number of possible predictors of LOS have been documented in prior studies, which include patient age, gender, diagnosis, severity of illness, response to treatment, physical comorbidities and psychosocial characteristics (Brownell & Roos, 1995; Jiménez et al., 2004). However, most of these studies have focused exclusively on the patients in psychiatric departments with no effort to examine the possible relationship between the use of physical restraints and LOS. Since it is recognized that the use of physical restraints may impose a harmful effect on patients' mental status, it is reasonable to hypothesize that the physical restraint use might lead to prolonged LOS.

To address this research gap, based on the medical records of 910 patients aged 60 or above admitted to a convalescent hospital in Hong Kong in 2007, this study explores the possible effect of physical restraint use on LOS after controlling for variables that have already been established as predictors of LOS. The results would provide evidence for ameliorating clinical practice such as fostering the sense of social support among nurses and implementing relevant institutional regulations so as to reduce the use of physical restraint use.

## 1. Method

This study adopted a retrospective design. The medical records of the selected patients admitted to Department of Medicine and Geriatrics of a public-funded hospital in Hong Kong in 2007 were recorded and studied. There were 521 beds for convalescence and rehabilitation in this hospital. The Department of Medicine & Geriatrics had 277 beds in 10 wards, providing multidisciplinary care in geriatric and stroke rehabilitation, and palliative care. The effect of physical restraint use on older patients' LOS was examined by using regression analysis.

### 1.1. Sample

Medical records of 1000 patients admitted to the Department of Medicine and Geriatrics of a publicly funded hospital in Hong Kong in 2007 were randomly selected from 3736 patients in the patient list with the aid of Statistical Package for Social Sciences (SPSS). To draw a random sample of 1000 at random from the patient list, we first clicked “Select Cases” from the “Data” menu, and we then selected “Random sample of cases” from the dialog box that emerged. Those who were discharged to other hospitals were excluded from this study.

### 1.2. Data collection

The records of patients aged 60 years and above in 2007 were extracted for the purpose of this study conducted during July to September 2011, hence a total of 910 medical records were included for further analyses. Three research assistants were responsible for reviewing the medical records and data entry. Information about the following variables that might affect LOS was obtained from each patient record: age, gender, accommodation before hospitalization, general health status, mobility, activities of daily living (ADL), mode of feeding, cognitive function, and the use of physical restraints. Although diagnosis and severity of illnesses have been recognized as variables that may affect LOS, it has always been a challenge to find a reliable and valid way to measure it, especially for patients with multiple pathologies (Jiménez et al., 2004). Considering that patients in this hospital for convalescence and rehabilitation purposes have multiple pathologies, their general health and functional status might be more important determinants of LOS and they could reflect the severity of illness of patients to some extent.

### 1.3. Measurement

All tools used in this study were validated in previous studies, and the reliability was vindicated by satisfactory Cronbach's alpha coefficients. The measurements used in this study were summarized as follows:

#### 1.3.1. Demographic characteristics

Demographic data included the patients' age, gender, and accommodation types before admission. Accommodation types were classified largely according to patients' family relationships. Patients either lived alone, lived with spouse only, lived with family members of two generations or more, or lived in old age homes. We further recoded them into living in old age home or not. In addition, patients' hospital number, ward admitted, dates of admission, and discharge were recorded.

#### 1.3.2. General health status and cognitive function

Patients' general health status was assessed by the physical condition domain of the Norton scale. The reliability and validity of the Norton scale were supported in a Hong Kong sample (Chan, Chow, French, Lai, & Tse, 1997). The scale is made up of five subscales, namely: physical condition, mental state, activity, mobility, and incontinence. Each scale has a rating from 1 to 4, with 1 representing the poorest clinical condition and 4 representing the best. Summing up the ratings on the five subscales yields a possible maximum score of 20. Patients are rated as “at risk” if they receive a score of 14 or below. The assessment using the Norton scale was usually conducted by registered nurses upon patients' admission. On the physical condition scale, patients were given a rating of either “very bad”, “poor”, “fair”, or “good”. Patients' cognitive function was assessed by the mental state domain of the Norton scale. In the present study, confused patients were indexed by having a score of 2 or below on the mental scale, and those who had a score of higher than 2 were considered as alert patients.

#### 1.3.3. Physical function

Patients' physical function was assessed in terms of their walking ability, ADL, and mode of feeding. Ambulation of patients was assessed by hospital physiotherapists using the Modified Functional Ambulatory Categories (MFAC) both upon admission and discharge (Holden, Gill, Magliozzi, Nathan, & Piehl-Baker, 1984). The functional ambulatory categories (FAC) is a 6-level scale to differentiate walking ability in terms of the extent of physical assistance needed. It has demonstrated very good test–retest and inter-rater reliability (Cohen's kappas of .95 and .91, respectively) (Mehrholz, Wagner, Rutte, Meissner, & Pohl, 2007). Slightly different from the FAC, the MFAC has a lowest classification of category 1 (bed bound) and highest of category 7 (independent outdoor walker). ADL of patients was assessed by occupational therapists using the Modified Barthel Index (MBI) (Shah, Vanclay, & Cooper, 1989). It comprises ratings on 10 areas of ADL, including: personal hygiene, bathing, feeding, toilet, stair climbing, dressing, bowel control, bladder control, ambulation, and chair/bed transfer. Summing up the 10 ratings, the MBI has a score which ranges from 0 to 100. According to the score, dependency levels are represented in five categories: total dependence (0–20), severe dependence (21–60), moderate dependence (61–90), slight dependence (91–99), and total independence (100). The MBI has shown an internal consistency of .90 (Shah et al., 1989).

With regard to patients' mode of feeding, types of diet were distinguished as: normal diet, soft diet, pureed diet, and tube feeding. This information was recorded in the discharge notes of each patient. In the cases where this information was absent in the discharge notes, such as when the patient died, case notes were reviewed where records of the patients' diet were documented.

#### 1.3.4. Use of physical restraints

In this study, the use of hand holder, safety vest, abdominal belt, seat belt, foot holder, table top, bedrail, or more than two restrainers were considered as the use of physical restraints on patients.

#### 1.3.5. Length of stay

It was calculated based on the number of days the patient stayed in the hospital according to the medical record.

### 1.4. Ethical considerations

The joint Chinese University of Hong Kong-New Territories East Cluster clinical research ethics committee approved the research protocol. All the information about the patients was kept in confidential and was used only for research purposes.

### 1.5. Data analysis

Data analyses were carried out with SPSS version 15. Descriptive analysis was first conducted, after which bivariate correlational analyses were performed between potential predicting factors and the dependent variable LOS. The Pearson's correlation analysis was run when the predicting factor was a scale variable, whereas the *η* correlation was used when the predicting factor was a nominal variable. To examine whether or not the selected variables (those who were significantly correlated with LOS) would retain their statistical significance in predicting LOS in a multivariate context, we further conducted hierarchical linear regression analysis. *P* < 0.05 was set to denote statistical significance.

## 2. Results

### 2.1. Characteristics of patients

As Table 1 shows, the 910 selected patients comprise three age groups: young old (60–69), mid-old (70–79), and old-old (80 and above), and most of them (90.1%) were in fair physical health status. Before the admission to the hospital, 636(69.9%) of them lived either by themselves, with spouse only, or with two or more generations; and only a small portion of patients (30.1%) lived in old age home. With regard to their mobility levels, 286 (32.3%) of them were either bed-bound or sitters; 448 (50.5%) needed companies of other people while walking; and the remainders were able to walk independently. Of the 910 participants, 53.7% were totally dependent or severely dependent in ADL; 34.7% were moderately dependent; and 11.6% were only slightly dependent or totally independent. In addition, 111 of them (12.2%) used tube feeding; 476 (52.5%) of them were on soft or pureed diet; and around one-third of them were on normal diet. Concerning their cognitive function, 621 (68.6%) of them were categorized as alert while the remainders were either categorized as stupor, confused, or apathetic. The data show that 122 (13.4%) of the patients have been physically retrained during hospitalization. Hand holder was most frequently used, followed by safety vest, abdominal belt, bed rail, table top, seatbelt, and foot holder. 41.8% of the restrained patients were restrained by more than one type of restrainers. The average LOS of the older patients was 19 days.

**Table 1.** Characteristics of patients.

Characteristics (Total *N* = 910)	Categories	*N* (%)/*M* (SD)
Age group	Young old (60–69)	76 (8.4)
	Mid-old (70–79)	328 (36.0)
	Old-old (80 and above)	506 (55.6)
Gender	Male	441 (48.5)
	Female	469 (51.5)
Accommodation before hospitalization	Alone	92 (10.1)
	With spouse only	147 (16.2)
	With two or more generations	397 (43.6)
	In old age home	274 (30.1)
General health status	Very bad	2 (0.2)
	Poor	22 (2.4)
	Fair	817 (90.1)
	Good	66 (7.3)
Mobility	Lyer	164 (18.5)
	Sitter	122 (13.8)
	Dependent walker	94 (10.6)
	Assisted walker	220 (24.8)
	Supervised walker	134 (15.1)
	Indoor walker	127 (14.3)
	Outdoor walker	26 (2.9)
Activities of daily living	Totally dependent (0–20)	187 (24.8)
	Severely dependent (21–60)	218 (28.9)
	Moderately dependent (61–90)	258 (34.2)
	Slightly dependent (90–99)	64 (8.5)
	Independent (100)	27 (3.6)
Mode of feeding	Tube feeding	111 (12.2)
	Pureed diet	196 (21.6)
	Soft diet	280 (30.9)
	Normal diet	320 (35.2)
Cognitive function	Stupor	62 (6.9)
	Confused	71 (7.8)
	Apathetic	151 (16.7)
	Alert	621 (68.6)
Use of physical restraints	Yes	122 (13.4)
	*(Hand holder)*	51 (41.8)
	*(Safety vest)*	23 (18.9)
	*(Abdominal belt)*	14 (11.5)
	*(Seat belt)*	2 (1.6)
	*(Foot holder)*	1 (0.8)
	*(Table top)*	7 (5.7)
	*(Bedrail)*	14 (11.5)
	*(More than one)*	51 (41.8)
	No	788 (86.6)
LOS in hospitals		19.1 (20.79)

Notes: “*M*”, Mean; “*N*”, number of patients; “SD”, standard deviation.

### 2.2. Correlations between potential predictors and LOS

Correlations between the nine potential predictors and LOS were calculated either by Pearson or *η* coefficients. The nine predictors included age, gender, living in old age home or not before hospitalization, mobility, ADL, mode of feeding, cognitive alertness, and the use of physical restraints. As Table 2 shows, there were no significant correlations between patients' demographic characteristics and their LOS. Although only weak correlations were observed, both their physical health and functional status (i.e. mobility, ADL, and mode of feeding) were found to be negatively correlated with their LOS, with statistical significance level reached *p* < 0.001. In addition, both cognitive alertness and absence of physical restraints were significantly related to shorter LOS (Table 2).

**Table 2.** Correlations between patient characteristics and their LOS.

Correlations	Coefficient (Pearson/*η*)	p*
Age and LOS	.007	ns
Gender and LOS	.017	ns
Old age home or not and LOS	.106	ns
General health status and LOS	−.141	***
Mobility and LOS	−.216	***
ADL and LOS	−.216	***
Mode of feeding and LOS	−.144	***
Cognitive function and LOS	−.237	***
Use of physical restraints and LOS	.116	**

Notes: “MFAC”, Modified Functional Ambulatory Categories; “MBI”, Modified Barthel Index; “LOS”, length of stay in hospitals.

***p < .001.

**p < .01.

*p < .05.

### 2.3. Hierarchical regression analysis of LOS

Based on the results of correlational analysis, hierarchical regression analysis with enter inclusion was conducted to examine the impact of the use of physical restraints on older patients' LOS after controlling for their general health condition, physical functioning, and cognitive alertness which were found to have significantly correlations with LOS in the bivariate context. In combination, general health status, physical and cognitive functioning, and the use of physical restraints explained 14% of the variance in LOS. As Table 3 shows, except for the mode of feeding (*β* = 0.05, *p* > 0.05), all other variables retained their statistical significance in the multivariate context. After controlling for the first block of variables, whether being physically restrained or not still acted as a potent predictor of LOS and it introduced a significant 2% increase in variance in LOS (*R* ^2^ change = 0.02, *F* = 12.87, *p* < 0.001). This suggests that the use of physical restraints exerts its effect independently of patients' general health status, physical and cognitive functioning status.

**Table 3.** Hierarchical regression analysis of LOS.

	Variables	
	Standard coefficient beta	
	Model 1 Δ*R*^2^ = 0.12	Model 2 Δ*R*^2^ = 0.02***
*General health status*		
Health condition	−.12**	−.12**
*Physical function*		
Mobility	−.15*	−.16*
ADL	−.23**	−.21**
Mode of feeding	.04	.05
*Cognitive function*		
Cognitive alertness	−.06*	−.09*
*Physical restraint use*		
Being physically restrained or not		.13***

Note: Δ*R* ^2^ = change of explained variance.

***p < .001.

**p < .01.

*p < .05.

## 3. Discussion

As expected, older patients' general health status, physical function, and cognitive function were found to affect their LOS significantly. This result is largely consistent with previous studies on the relationship between patient characteristics and LOS (Brownell & Roos, 1995; Jiménez et al., 2004; Maguire, Taylor, & Stout, 1986). More importantly, after controlling for patient characteristic factors, the use of physical restraints on patient was also found to be predictive of longer LOS of older patients. The results that restrained older patients tended to have longer LOS is consistent with the findings of previous observational studies (Evans, Wood, & Lambert, 2003; Frengley & Mion, 1986; Mion, Frengley, Jakovcic, & Marino, 1989; Robbins, Boyko, Lane, Cooper, & Jahnigen, 1987). As these studies were conducted more than two decades ago, the present study has added more empirical evidence for the positive relationship between physical restraint use and patients' LOS.

A plausible explanation to this finding may be related to the adverse effects of physical restraint use. It is evidenced in the previous literature that physical restraint use might lead to a number of negative outcomes, including nightmares, feelings of powerlessness, and even agitated behaviors (Chien et al., 2005; Lin et al., 2008; Mohr & Pumariega, 2001). It has been criticized that these negative outcomes may interfere with treatment progress of restrained patients (Mott, Poole, & Kenrick, 2005). In addition, restraint use may result in later mobilization for those patients who need mobilization earlier. Consequently, this may defer the conditions for which the patients are fit to be discharged and prolong the stay. As a matter of fact, LOS can be seen as an indicator of efficiency of inpatient care and bed use (Brownell & Roos, 1995; Jiménez et al., 2004), the findings provide a potential direction for enhancing hospital service by improving bed planning and quality of care.

In addition, shortened LOS may relieve the financial burden of both families and the government, and achieve more effective resource utilization. According to the latest survey report by the Census and Statistical Department, public hospitals in Hong Kong accommodate for more than 78% of the admission cases (Census and Statistic Department, 2010). In the fiscal year 2004/2005, the total expenditure on public inpatient care cost more than HKD 21 billion (Food and Health Bureau, 2008). With ever increasing load and demand from the public on medical service provision, it is of significant importance that the hospitals operate in an efficient and cost effective manner. The results of the present study therefore shed light on seeking manageable ways that might benefit hospital administration by reducing the use of physical restraints on older patients.

### 3.1. Limitations

Several limitations of this study must be acknowledged. First, this retrospective study used a cross-sectional design rather than a longitudinal one. Thus, the causal relationship between the use of physical restraints and older patients' LOS is not warranted and needs further examination. Second, although the regression model of LOS, which used patients' general health status, physical functioning, cognitive functioning, and physical restraint use as independent variables, could be claimed to be an effective model in predicting LOS, they only explained 14% of variance in LOS. This might be either because that we neglected some other potent determining factors of LOS in the model, or be owing to the crude measurement of general health status, and cognitive function. The model that we developed in this study focused more on the health and functional status of patients, it is desirable to further investigate how factors such as marital status and social support may affect LOS. Third, the fact that hand mitten was not properly documented as one type of hand restraints, and that the use of bedrail seemed to be under reported, may bias the findings.

## 4. Conclusion and recommendations

Despite these limitations, to the best of the authors' knowledge, the present study is the first of its kind which systematically examined the effects of using physical restraints in hospital setting on older patients' LOS by adopting a retrospective design. The results indicated that older patients' general health status, physical function, and cognitive function were important factors affecting their LOS. In addition, independent of these factors, the use of physical restraints was still found to be significantly predictive of older patients' LOS, and these two blocks of variables together served as an effective model in predicting LOS.

Generally speaking, we would suggest that physical restraint should be used with more caution and the use of it should be reduced on older patients in the hospital caring setting. By addressing the limitations identified in this study, we may further develop some programs on physical restraint reduction and examine their resultant outcomes using a longitudinal design. Since nurses' knowledge and attitude toward physical restraint use have been found to affect the frequency of restraint use (Karlsson, Bucht, Eriksson, & Sandman, 2001; Pellfolk et al., 2010), we would recommend that measures to be taken to provide thorough education to relevant medical staff and nursing students on the use of physical restraints and other desirable clinical practice (Smith & Barry, 2012). Meanwhile, hospital managers should develop strategies to utilize the distributional characteristics of nursing staffs among hospital wards. For example, hospital managers may avoid the situation where less experienced nurses cluster in certain wards by placing experienced nurses evenly among hospital wards. Alternatively, wards that are prone to use physical restraints (e.g. geriatric wards) may be stationed by more experienced nurses than inexperienced ones. In addition, nurses should be encouraged to make compassionate, morally sound, and technically reasonable decisions about the treatment of patients.

According to our data, hand holders were the most frequently used restraint for patients while the main reason for the use of hand holders was to secure tube feeding. Thus, it is natural to expect that the use of hand holders can be effectively reduced if the health-care team can assess the need for continuation of tube feeding more frequently, and more effort can be made in hand feeding rather than simply using nasogastric tube feeding. When the use of restraints is unavoidable, especially in some cases requiring long-term tube feeding, percutaneous endoscopic gastrostomy can be used which may be better tolerated by patients, and the hand restraint can be made more comfortable by using hand mittens.
